# Sensory Evaluation of Locally-grown Fruit Purees and Inulin Fibre on Probiotic Yogurt in Mwanza, Tanzania and the Microbial Analysis of Probiotic Yogurt Fortified with *Moringa oleifera*

**Published:** 2015-03

**Authors:** Sharareh Hekmat, Kathryn Morgan, Mohammad Soltani, Robert Gough

**Affiliations:** ^1^Division of Food and Nutritional Sciences, Brescia University College, 1285 Western Rd. London, Ontario, N6G 1H2, Canada; ^2^The University of Western Ontario, 1285 Western Rd. London, Ontario, N6G 1H2, Canada

**Keywords:** Avocado, Inulin, *Moringa oleifera*, Probiotic yogurt, Sensory evaluation, Tanzania

## Abstract

The purpose of this study was to establish new food products that increase the nutritional value and health benefits of the probiotic yogurt currently used in the Western Heads East (WHE) Project in Mwanza, Tanzania. The probiotic yogurt has established health benefits, and product development through fortification must not adversely affect the acceptability of yogurt or the viability of the probiotics. Both sensory testing and microbial analysis testing were conducted. The products tested were yogurt fortified with locally-grown fruit purees with inulin and *Moringa oleifera.* The results of the sensory evaluation showed that all yogurts were not rated significantly different from the control, except for appearance. The avocado puree without inulin rated significantly lower in all categories. The microbial analysis showed that *Moringa oleifera* did not negatively affect the growth of *Lactobacillus rhamnosus* GR-1 in MRS, milk or yogurt, although a significant decrease was found after 5 weeks of storage at 4 ^o^C.

## INTRODUCTION

According to the United Nations Development Programme (UNDP), HIV has inflicted the “single greatest reversal in human development” in modern history (1). For this reason, the research and development of products that can contribute to the health and economic vitality of a community is crucial. Probiotics, meaning “for life”, are “live micoorganisms, which when consumed in adequate amounts, confer a health effect on the host” (2). A study by Anukam *et al.* (2008) showed an increase in cluster of differentiation 4 (CD4) cell counts, a marker of immune status, resulting in a 3 to 4-fold improvement for those receiving *Lactobacillus rhamnosus* GR-1 and *L. reuteri* RC-14 compared to controls (3), with further confirmation of their effects in a study by Irvine *et al.* (4). *L. rhamnosus* GR-1 is generally stable over its shelf-life (5). This strain has been studied extensively in milk products, including yogurt.

Probiotic yogurt is maintaining its popularity as a functional food (6) and is an excellent vehicle for fortification, through which individuals can receive greater health benefits above those listed previously. Micronutrients, such as those found in fruits, can be inexpensive ways to increase the nutritional value of the yogurt. Fruits commonly found in East Africa can be the acceptable sources of these additional nutrients. Due to high rates of malnutrition, particularly concerning micronutrients, this is where product development can have the greatest impact on the health of consumers in the Global South. Broad-spectrum micronutrient interventions, normally in capsule form, have been associated with delayed progression of HIV to AIDS, improved immune function by increased CD4 and delayed HIV-related mortality (7–10).

Inulin is a naturally-occurring carbohydrate in a variety of plants, the most commonly used is chicory root. Oligo-fructose can be formed through partial hydrolysis of inulin, and both are considered functional fibres (11). Naturally-occurring fructans are found in onions, bananas, wheat, garlic, and many other whole foods (11,12). These elements of the diet are considered prebiotic and are defined as “food ingredients that promote the growth or activity of a limited number of bacterial species for the benefit of host health” (12). Therapeutic dosage in some reported studies were shown to range from as low as 3 g/day to 10 g/day (11,12). The substantiated claims of inulin are that it acts as a bulking agent, can beneficially promote healthy colonic microflora, increase calcium and magnesium absorption in the gastrointestinal tract, and reduce hepatic lipogenesis resulting in improved lipid homeostasis (11,12). There appears to be an immunostimulation effect by inulin through targeting gut-associated lymphoid tissues (GALT). Fermentation of prebiotics in the bowel provide short-chain fatty acids to colonocytes, promoting a healthy gut, in turn, normalizing bowel function, improving colon integrity, colonization resistance, and, thus, contributing to the health of the individual (12). The sensory properties of inulin that could be utilized in the development of a yogurt product include its contribution to overall food quality through texture modification. It has also been shown to prolong shelf-life and maintain quality scores for taste, flavour, odour, and overall acceptability over storage time as well (11).

The second focus of this study was to utilize *Moringa oleifera.* This plant species is commonly used in countries, such as India, Africa, Southeast Asia, South America, and the Pacific and Caribbean Islands. It has been consumed in diverse culinary ways with a reputation for its nutritional values in addition to having medicinal properties (13). *M. oleifera* contains various fatty acids comprising oleic, palmitic, steric and behenic acids. The leaves contain vitamin A, C, and E as well as their provitamins. These nutrients are known to sequester free radicals when combined with a balanced diet and may have immunoprotective effects (14). Other reports show that the leaves contain high amounts of total phenols and proteins and are a good source of calcium, potassium, magnesium, iron, manganese, and copper, all of which are essential to the diet (15). The World Health Organization has emphasized its use as a low-cost supplement for the poorest countries around the world (1,16).

The purpose of this study was to establish new food products that increase the nutritional value and health benefits of the probiotic yogurt used in the Western Heads East Project (Mwanza, Tanzania). The probiotic yogurt has established health benefits; therefore, product development through fortification must not adversely affect the acceptability of yogurt or the viability of the probiotics. Both sensory testing and microbial analysis testing were conducted.

## MATERIALS AND METHODS

### Preparation of probiotic culture

*Lactobacillus rhamnosus* GR-1 (Urex Biotech Inc., London, ON) probiotic mother culture was prepared at the NIMR laboratory facilities in Mwanza, Tanzania. The probiotic microogranisms were added directly to sterilized de Man, Rogosa and Sharp (MRS) broth and incubated anaerobically, using BBL gas pack (Becton, Dickinson and Company, Sparks, Maryland, USA) at 37 °C overnight. Five hundred mL of fluid milk (3.5% fat) containing 0.4% yeast extract (Becton^TM^) was inoculated with 1% probiotic broth mixture. The inoculated milk was then incubated anaerobically at 37 °C for 18 hours, and then stored at 4 °C in an airtight container prior to pick-up.

### Preparation of probiotic yogurt

The yogurt samples were prepared by heating standardized (3.25% fat) milk to 85 °C and holding it for 30 minutes at this temperature. After letting it to cool to 37 °C, 4% of the prepared probiotic culture and 2% of regular yogurt for the starter culture, which contained *Lactobacillus delbruekii subsp. bulgaricus* and *Streptococcus thermophilus*, were used in order to inoculate the milk. This was incubated for 6 hours at 37 °C, and then stored in a refrigerator.

### Preparation of samples with inulin and local fruit purees

Four teaspoons (3.2 g per tsp) of powdered Oliggo-Fiber^®^ inulin (VWR-International, Mississauga, Ontario, Canada) were added to each of the 1 litre prepared yogurt samples for a total of 3 samples containing the prebiotic. Oliggo-Fiber^®^ inulin is an unflavoured powder derived from chicory root. Each 200 g serving of yogurt contained 2.56 g, which when combined with other foods in the diet could provide a therapeutic amount of prebiotic (17). Food ingredients were selected for incorporation into the probiotic yogurt based on the availability and evidence of therapeutic functionality (18,19). A banana puree was prepared using 7 medium-sized bananas (118 g each). The pureed bananas were divided between 2 samples of yogurt (1 L), one containing the prebiotic and one without. Table 1 shows the nutrient content profile of the banana puree per 200 g serving of yogurt. An avocado puree was prepared using 3 large ripened fruits (201 g each). The avocados were combined with 6 teaspoons of sugar to add flavour. This mixture was divided between 2 samples of yogurt (1 L), one containing the prebiotic and one without. The prebiotic and puree (banana and avocado) were mixed thoroughly to ensure even distribution throughout the yogurt product. Table 2 shows the samples that were used in the sensory evaluation.

### Preparation of ***Moringa oleifera***-supplemented MRS broth and milk

*Trial A:* Three formulations of MRS-based test tubes were prepared. MRS broth was prepared using 5.2% by weight of MRS powder dissolved into de-ionized water. Each test tube contained 10 mL of MRS broth combined with 0%, 1% (0.1 g), and 5% (0.5 g) of crushed dried leaves (6-8 months old) of *M. oleifera*, which were then autoclaved at 15 PSI for 15 minutes. Once the test tubes were cooled to 37 °C, 1 mL of pipetted prepared probiotics was added to each. The test tubes were sterilized at the tip, using a bunsen burner and covered with aluminum foil, then anaerobically incubated (using BBL gas pack) at 37 °C overnight. Three more formulations were prepared using the same method; however, 2% milk was substituted for the MRS base. Table 3 shows the nutrient formulations for Trial A.

**Table 1.** Nutrient content of banana puree, avocado puree, and *Moringa oleifera* (powdered) per 200 g serving of yogurt

Nutrient	Banana	Avocado	Moringa
Kilocalories	73.6	96.8	2.05
Protein (g)	0.88	1.2	0.271
Fat (g)	0.24	SFAs^a^-1.28 MUFAs^b^-5.92 PUFAs^c^-1.12	0.023
Carbohydrate (g)	18.88	5.12	0.382
Fibre (g)	2.16	4.08	0.192
Sugar (g)	10.08	0.4	-
Calcium (mg)	4.2	7.2	20.03
Iron (mg)	0.24	0.32	0.282
Magnesium (mg)	22.4	17.4	3.68
Phosphorous (mg)	22.75	39.4	2.04
Potassium (mg)	123	365.6	13.21
Sodium (mg)	0.72	4.24	-
Zinc (mg)	0.16	0.4	-
Vitamin C (mg)	7.2	6	0.173
Folate (dietary folate equivalents) (mcg)	16.8	48.88	-
Vitamin A (retinol activity equivalents)	2.8	4.24	13.58
Vitamin E (alpha-tocopherol) (mg)	0.08	1.36	1.13
Thiamin (mg)	0.024	0.04	
Riboflavin (mg)	0.064	0.08	0.026
Niacin (mg)	0.56	1.04	0.082
Vitamin B6	0.4	0.2	
Vitamin B12	0	0	

^a^Saturated fatty acids

^b^Mononusaturated fatty acids

^c^Polyunsaturated fatty acids

**Table 2.** Probiotic yogurt containing food additives for sensory panel testing

Sample	Food additive
1	Plain yogurt-(Control)
2	Plain yogurt + Inulin
3	Avocado + Inulin
4	Avocado
5	Banana + Inulin
6	Banana

**Table 3.** Nutrient formulations for Trial A

Sample	Medium
1	MRS-1% Moringa*
2	MRS-5% Moringa*
3	MRS-Control*
4	Milk-1% Moringa*
5	Milk-5% Moringa*
6	Milk-Control*

*All formulations contained 1% probiotic culture

### Preparation of ***Moringa oleifera***-supplemented yogurt

*Trial B:* Standard probiotic yogurt was prepared as described previously. There were five different formulations prepared. One yogurt was the control without any modification. Two formulations contained 5% sugar by weight and 0.5% and 1% *M. oleifera* by weight. The other two formulations contained the same percentage by weight of *M. oleifera* without the addition of sugar. For the samples containing sugar—Sample 2 and 3 (Table 4)—this ingredient was added prior to heating the milk to 85 °C for 30 minutes. *M. oleifera* was added during the cooling process to 37 °C prior to the inoculation of the probiotic and starter yogurt cultures into the milk. The nutritional value per serving of 200 g of yogurt is shown in Table 1.

**Table 4.** Nutrient formulations for Trial B

Sample	Medium
1	0.5% *Moringa* + 5% Sugar
2	1% *Moringa* + 5% Sugar
3	0.5% *Moringa*
4	1% *Moringa*
5	Yogurt-Control

All yogurt formulations contained 4% probiotic culture and 2% starter culture

### Selection of subjects and sensory evaluation

To be eligible to participate in this study, panelists had to be over the age of 16 years, had to have the capacity to understand the intent of the study, and had to make an informed decision regarding consent. Twenty-five untrained volunteer panelists were recruited through convenience sampling in the Mabatini district kitchen (Mwanza, Tanzania) and asked to evaluate the 5 samples of probiotic yogurt containing a locally-grown fruit puree with or without Oliggo-Fiber^®^ inulin. Participants ranged from 16 to over 50 years of age and were assisted by the WHE coordinator, to interpret the questions of the study, if required. Upon completion of the study, participants were given a voucher for the purchase of 500 mL probiotic yogurt from the kitchen.

Sensory evaluation of the yogurt samples took place in the community yogurt kitchen in Mwanza, Tanzania, specifically the district of Mabatini. The panelists completed their evaluations during their scheduled appointment times between 10:00 am and 4:00 pm. The purpose of the study was explained to the panelists through a letter of information with the assistance of the coordinator, if required, and the panelists signed an informed consent form if they were in agreement with the purpose of the study. The researcher collected the signed consent forms. Participants were informed that the probiotic yogurt contained either pureed avocado or banana and, possibly, inulin fibre.

Evaluations were conducted individually and in a quiet area of the yogurt kitchen. Panelists were asked not to communicate with each other until the evaluation was complete. Each participant was given six samples of yogurt in random order, with a random 3-digit code on the cup, an evaluation form (translated to Kiswahili), a pen, and a cup of water, which they were instructed to rinse their mouth between each tasting. Yogurt samples were placed in uniform plastic cups and served with a separate plastic spoon to ensure no carryover of sensory properties (20). Panelists were given uniform amounts of each sample—approximately 45 mL—and were asked to evaluate the appearance, texture, flavour, and overall acceptability of each sample on a nine-point hedonic scale in which 1=dislike extremely, 5=neutral, and 9=like extremely. Further questions were asked to explain their evaluations. Both ratings and comments were recorded on the evaluation form. The University of Western Ontario Research Ethics Boards for Human Subjects and the Brescia University College Ethical Review Committee approved this study.

### Microbial analysis

A 0.85% saline solution was prepared, and 99 mL of this solution was measured into bottles. The dilution bottles were then sterilized using an autoclave at 15 PSI for 15 minutes. The bottles were removed and cooled overnight. In Trial A, the formulations were diluted to 10^6^ and 10^7^. In Trial B, the formulations were diluted to 10^5^ and 10^6^. Diluted sample of 0.1 mL was pipetted onto selective MRS agar plates containing 15 μg/mL fusidic acid to enumerate the *L. rhamnosus* GR-1 probiotic strain. Microbial analysis for both trials took place over 5 weeks. Samples were plated and counted at Week 1, 3, and 5 to test the bacterial viability over the storage period at 4 ^o^C.

### Statistical analysis

Sensory evaluation and microbial counts were analyzed using SPSS 2.0 software. An ANOVA was used in order to test the overall significance, and Tukey's adjustment tests for multiple comparison were applied.

## RESULTS

### Sensory properties

There were significant differences in appearance of the yogurt samples. The overall result of ANOVA, comparing the appearance of the yogurts, was significant (p<0.0001). Pairwise testing, using Tukey's adjustment for multiple testing, found the plain yogurt with and without inulin (Sample 1 and 2) to have a mean score of 7.04 compared to 4.76 for the avocado without inulin (Sample 4) at the significance level of p=0.0018. Sample 1 and 2 were significantly higher than the means for yogurt Sample 3 (p=0.0095), 5 (p=0.0404), and 6 (p=0.0182) (Table 5).

There were significant differences in the overall flavour profile of all yogurt samples (p=0.0373). The pairwise differences (Tukey's adjustment for multiple testing) were significant between yogurt Sample 4 and 5 (p=0.0127). Only the difference between yogurt Sample 4 and 5 was statistically significant. The mean for yogurt Sample 4 was 4.72, and for Sample 5, was 6.84—the largest pairwise difference. The banana puree combined with inulin (Sample 5) had a significantly more favourable flavour than the avocado without inulin (Sample 4).

The score for texture ranged from 6.6 (Sample 1) for the control to a low of 4.72 for the avocado puree (Sample 4) with p=0.0229. The other samples ranged from 5.24 to 6.12, which would correspond to a rating of “neither like nor dislike” to “like slightly” on the 9-point hedonic scale.

Overall acceptability for all yogurt samples was not found to be significantly different between the various types. The lowest score (4.92) for the avocado without inulin (Sample 4), compared to the highest mean score of 6.32 for both plain yogurt with and without inulin (Sample 1 and 2) (p=0.2582), had the greatest difference (Table 5).

### Viability of ***L. rhamnosus*** GR-1 in ***Moringa oleifera***-supplemented yogurt

To confer health benefits, the bacterial colony formations cannot fall below 10^6^ CFU/mL (11). No scores fell below 3×10^7^ CFU/mL in Trial A and 0.9×10^7^ CFU/mL in Trial B (Table 6), although a few plates had no visible colonies formed and were, therefore, unable to be counted. The CFU count remained high despite a significant drop from Week 1 to Week 5 in Trial A (p≤0.0001) and Trial B (p=0.0058) when analyzed using a log growth scale for the mean bacterial count. In Trial A, the MRS control (Table 3) was significantly lower than Sample 1 (p=0.0210), 2 (p=0.0011), 4 (p=0.0007), and 6 (p=0.0215). The best growth of *L. rhamnosus* GR-1 was under the milk conditions at 1% *Moringa*. In Trial B, the control yogurt (Table 4) showed the best results for growth, which was significantly higher than yogurt with the highest concentration of *Moringa* at 1% (Sample 3) and without sugar (p=0.0219). The other samples were not significantly different from the growth seen in Sample 5 and Sample 3.

**Table 5.** Sensory characteristics (mean score ± standard deviation)

Sample	Appearance	Flavour	Texture	Overall
1	7.04±2.01	6.08±2.38	6.60±2.06	6.32±2.39
2	7.04±2.01	5.80±2.65	6.12±2.66	6.32±2.54
3	5.04±2.44	6.24±1.92	5.24±2.28	5.44±2.35
4	4.76±2.26	4.72±2.11	4.72±2.34	4.92±2.50
5	5.32±2.67	6.84±2.07	5.68±2.56	6.12±2.70
6	5.16±2.43	5.96±2.91	5.32±2.44	6.12±2.85

**Table 6.** Microbial analysis of Trial A and Trial B

Sample	Trial A (CFU/mL)	Trial B (CFU/mL)
1	4.2×10^7^	1.78×10^7^
2	4.78×10^7^	1.79×10^7^
3	3.24×10^7^	1.17×10^7^
4	4.86×10^7^	0.9×10^7^
5	3.38×10^7^	2.08×10^7^
6	4.21×10^7^	NA

NA=Not available

## DISCUSSION

Creating functional food products is becoming increasingly attractive, with the potential to really impact the devastation of malnutrition in the Global South. In particular, malnutrition is a major complication of HIV infection (21). Malnutrition is prevalent due to infection, decreased caloric intake, malabsorption, increased energy needs, and opportunistic infections, resulting in decreased CD4 count leading to a weakened immune system diminishing the ability to respond to therapeutic treatment. Malnutrition is a significant predictor of survival rate for PLHAs (21–23). Lower plasma levels of vitamin A, E, B12 relating to faster disease progression and micronutrient deficiencies are also independently linked to low CD4 cell counts (a measure of immune status), advanced HIV-related diseases, faster disease progression, and HIV-related mortality (7,24,25).

Hemsworth cited that external fortification of nutrients would be considered unsustainable (26). However, using locally-grown fruits, such as bananas and avocados, may be beneficial as it can improve fruit consumption in regions or populations where traditionally it may be low, thereby adding nutrient values to their diet without adding excessive costs. Secondary to the health benefits, there would be an increase in flavour, appearance, and overall acceptability of the yogurt, which may promote more regular consumption. Regular consumption is particularly important for probiotics to work effectively (5,17). Inulin is an unflavoured ingredient, and its incorporation has not been found to alter the mean appearance or texture scores in previous studies (27,28). This was found to be true in this study as well. For the avocado sample, the presence of inulin may have resulted in the better overall impression of the yogurt. The overall appearance was significantly lower in the yogurts that had purees added to them with and without the inulin. This may be due to an increased lumpiness. If this is the reason for a lower score, it could be improved through better processing methods. The puree was created by mashing the fruits but could alternatively be pureed using an electric machine. However, due to the lack of availability of an appliance and also due to the inconsistent availability of electricity often experienced in this region, an electric pureeing-machine was not used. Only the avocado without inulin rated slightly below 5, which corresponded to the scale of neither like or dislike.

The avocado puree without inulin performed the poorest in all 4 areas of the sensory evaluation, perhaps suggesting that the inulin played a role in improving the profile of the avocado-based samples in some respects. Both puree and inulin additions could be used without significantly altering the sensory characteristics. Regular consumption of these products would receive both health benefits from the additional nutrients and functional benefits for the gastrointestinal tract and metabolism (17).

Despite *Moringa* being used widely in many regions around the world, concerns over the use of *M. oleifera* seeds to act as an anticyanobacterial agent warranted further investigation for its use in a food product containing bacteria, of which the survival is of the utmost importance in order to remain in viable amounts to confer health benefits to the host (29). In a study by van Tienen *et al.*, the growth of the probiotics in *M. oleifera-*supplemented yogurt was found to have a growth-enhancing effect (30). The results found in this study suggested that *Moringa* did not inhibit the growth of bacteria, except perhaps in higher doses that did not contain sugar. Sugar may have countered any antibacterial properties *Moringa* has at larger quantities as it would be an additional food source for them. Alternatively, the MRS broth with the 5% *Moringa* in Trial A had significantly greater growth than the control, which may suggest growth-enhancing properties and could be further tested for prebiotic properties.

In-house testing for the *Moringa*-fortified yogurt suggested that, a level of 0.5% *Moringa* with 5% sugar was acceptable as yogurt. Also, it was suggested that *Moringa* without sugar at 0.5% could be acceptable as a dip with a good flavour. At 1% *Moringa*, the yogurt samples had a strong undesirable flavour. Further research would need to assess the *Moringa* yogurt at 0.5% with sugar for acceptability in the East African communities in which it would be consumed. Alternatively, the 0.5% *Moringa* without sugar could be tested as a dip on an appropriate serving medium, such as *chapati*, locally-consumed flat bread, and unleavened bread. Both of these products had never been tested in these forms. van Tienen *et al.* previously tested *Moringa* in greater concentrations at approximately 0.854% and 1.709% and without added sugar or as a form of dip and did find an acceptable product (30). Although 0.5% would not satisfy meeting the criteria for a ‘good source’ of vitamin A as suggested in this paper, it would be a source of nutrients and could potentially improve the acceptability of the *Moringa*-fortified yogurt or dip.

### Conclusions

Future testing, if either of these products are found to be acceptable, would be to determine the biochemical profile of recipients to see if the micronutrients are in quantities great enough to promote improved lab values for serum vitamin A, E, B and have an effect on increasing the CD4 levels above those achieved through the health benefits of probiotic yogurt alone.

## ACKNOWLEDGEMENTS

This study was supported by Western Heads East Project affiliated with The University of Western Ontario.

**Conflict of interest:** Authors declare no competing interests.
